# Patterns of First-Line Systemic Therapy Delivery and Outcomes in Advanced Epithelial Ovarian Cancer in Ontario

**DOI:** 10.3390/curroncol29080472

**Published:** 2022-08-22

**Authors:** Shiru L. Liu, Wing C. Chan, Geneviève Bouchard-Fortier, Stephanie Lheureux, Sarah E. Ferguson, Monika K. Krzyzanowska

**Affiliations:** 1 Institute of Health Policy, Management, and Evaluation, University of Toronto, Toronto, ON M5T 3M6, Canada; 2 Department of Medical Oncology, BC Cancer-Surrey, Surrey, BC V3V 1Z2, Canada; 3 ICES, Toronto, ON M4N 3M5, Canada; 4 Division of Gynecology Oncology, University Health Network (UHN)/Sinai Health System, Toronto, ON M5G 2C1, Canada; 5 Department of Obstetrics and Gynecology, University of Toronto, Toronto, ON M5G 1E2, Canada; 6 Division of Medical Oncology, University Health Network (UHN), Toronto, ON M5G 2C1, Canada

**Keywords:** ovarian cancer, bevacizumab, real-world evidence, health care utilization

## Abstract

Background: First-line treatment of epithelial ovarian cancer (EOC) consists of a combination of cytoreductive surgery and platinum-based chemotherapy. Recently, targeted therapies such as bevacizumab have been shown to improve oncologic outcomes in a subset of a high-risk population. The objective of this study is to evaluate the patterns of practice and outcomes of first-line systemic treatment of advanced EOC, focusing on the adoption of bevacizumab. Methods: A population cohort study was conducted using administrative data in Ontario, Canada. Patients diagnosed with advanced stage non-mucinous EOC between 2014 and 2018 were identified. Datasets were linked to obtaining information on first-line treatment including surgery, systemic therapy, providers of care, systemic therapy facilities, and acute care utilization (emergency department (ED) visits and hospitalizations) during systemic treatment. Multivariate logistic regression was used to determine factors associated with systemic therapy utilization. Results: Among 3726 patients with advanced EOC, 2838 (76%) received chemotherapy: 1316 (47%) received neoadjuvant chemotherapy, 1060 (37%) underwent primary cytoreductive surgery followed by chemotherapy, and 462 (16%) received chemotherapy only. The median age was 67 (range: 20–100). Most chemotherapies were prescribed by gynecologic oncologists (60%) and in level 1 academic cancer centres (58%). Only 54 patients (3.1%) received bevacizumab in the first-line setting after its approval in Ontario in 2016. Bevacizumab was more likely to be administered by medical oncologists compared to gynecologic oncologists (OR 3.95, 95% CI 2.11–7.14). In total, 1561 (55%) and 1594 (56%) patients had at least one ED visit and/or hospitalization during systemic treatment, respectively. The most common reasons for ED visits were fever and bowel obstruction. Conclusion: Patterns of care for EOC in Ontario differed between care providers. The uptake of bevacizumab for first-line treatment of EOC was low. Acute care utilization related to EOC was high.

## 1. Introduction

Epithelial ovarian cancer (EOC) is the leading cause of death among gynecologic malignancies [1]. Most EOC consists of high-grade serous carcinomas (HGSC), which are commonly diagnosed at an advanced stage, i.e., FIGO (Fédération Internationale de Gynécologie et d’Obstétrique) stage III or IV. The estimated five-year overall survival for EOC is approximately 46%, and the prognosis is worse for those with stage IV disease at presentation or other high-risk features such as unresectable disease or suboptimal cytoreductive surgery [2].

Standard first-line treatment for advanced EOC consists of a combination of platinum-based chemotherapy and cytoreductive surgery, with the goal of achieving no residual disease. Chemotherapy can be delivered in the adjuvant and/or neoadjuvant settings, depending on the extent of the disease at the time of presentation [3]. In addition, intraperitoneal (IP) chemotherapy can be considered for those with optimally cytoreduced stage III/IV disease after primary surgery [4]. Clinical outcomes have improved with the emergence of targeted therapies such as bevacizumab [5,6], an anti-angiogenesis agent, for a subset of ovarian cancer patients, and more recently, polyadenosine diphosphate-ribose polymerase (PARP) inhibitors [7]. 

Bevacizumab was the first targeted therapy approved for ovarian cancer in both the first-line and recurrent settings [8,9,10]. The landmark ICON 7 trial demonstrated an improvement in progression-free survival (PFS) of 6 months for EOC patients treated with combination chemotherapy and bevacizumab, along with maintenance bevacizumab, and overall survival (OS) of 10 months in a pre-specified subgroup of patients with high-risk features, such as stage IV, unresectable disease or residual disease at the end of surgery [5]. A recent updated systematic review and meta-analysis confirms improvement in PFS for bevacizumab combination therapy in the first-line setting for patients with high-risk features treated with chemotherapy and bevacizumab combination [11]. Since April 2016, bevacizumab in combination with chemotherapy has been approved and funded in the province of Ontario, Canada, for high-risk advanced EOC in the first-line setting [12]. However, its uptake in the real world is unknown, and clinical factors associated with its use and toxicity have not been well documented.

Currently, there is considerable heterogeneity in the management of these patients across North America due to variations in public policy and funding [13], institutional infrastructure, which can influence referral patterns [14], and physician specialization [15]. With increasing approval and use of targeted therapies, it is important to understand the uptake of such treatment and to explore factors that may lead to differences in care, and ultimately, differences in outcomes. The main objective of this study was to evaluate the pattern of first-line treatment of advanced EOC in Ontario, focusing on the uptake of bevacizumab combination therapy among different care providers and institutions. The secondary objectives were the following: (1) to assess acute care utilization during treatment, including emergency department (ED) visits and hospitalizations, focusing on those treated with bevacizumab combination therapy; (2) to determine overall survival.

## 2. Materials and Methods

### 2.1. Data Sources

This study was a provincial, population-based retrospective cohort study using linked administrative databases held at ICES (formerly known as Institute of Clinical and Evaluative Sciences), a non-profit research organization, which collects health-related information on Ontario residents for purposes of improving health care. The linked datasets that were used included the following: Ontario Cancer Registry (OCR), Ontario Health Insurance Plan (OHIP), Registered Persons Database (RPDB), Activity Level Reporting (ALR), New Drug Funding Program (NDFP), Ontario Drug Benefit (ODB), ICES Physician Database (IPDB), and Canadian Institute of Health Information (CIHI) databases (Appendix A Table A1).

The study protocol was approved by the Research Ethics Board at University Health Network.

### 2.2. Cohort Creation

**Figure A1:**
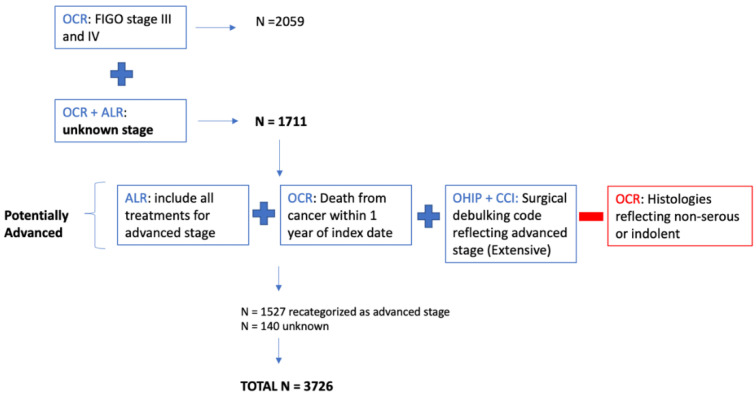
Algorithm to determine potentially advanced stage for unknown stage category.

All adult women with a diagnosis of ovarian, fallopian tube, or primary peritoneal cancer (Appendix B Table A2) between 1 January 2014, and 31 December 2018, were identified from the OCR. The most common non-mucinous histologies were included to reflect high-grade disease (Appendix B Table A3). Mucinous histology was excluded due to its unique biology and treatment mirroring that of gastrointestinal malignancies. Other exclusions were age < 18, non-Ontario residents or OHIP ineligible on their cancer diagnosis date, those with a previous diagnosis of non-cutaneous malignancy within 5 years of ovarian cancer diagnosis, and those with early-stage disease (stage 0, I, II). For patients with missing stage information, listed as unknown, we developed an algorithm to identify patients likely to have advanced disease based on treatment and surgical codes (Appendix C Figure A1). 

**Figure A2:**
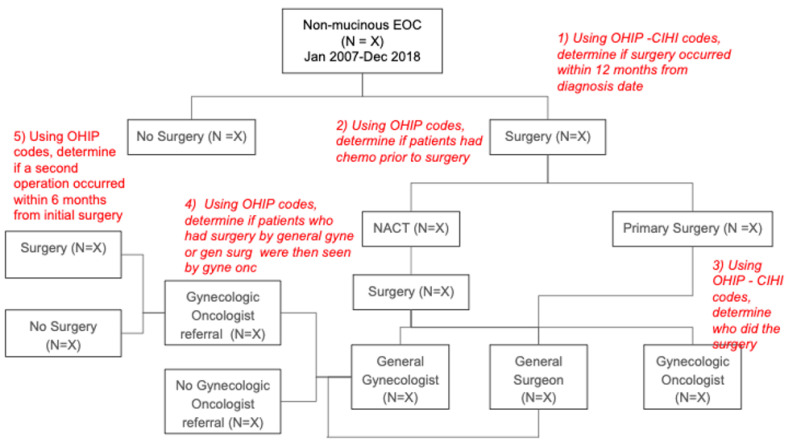
Initial cohort creation algorithm. Adapted from G. Bouchard-Fortier data creation plan (initial cohort).

Ovarian cancer surgery was identified using surgical codes from OHIP and CIHI’s Canadian Classification of Health Interventions (CCI) (Appendix D Figure A2). In consultation with gynecologic oncologists, surgical codes, which would reflect multi-visceral cytoreductive surgery in advanced-stage disease, including those representing extensive bowel surgeries, were selected (Appendix D Table A4).

### 2.3. Treatment Cohorts

**Figure A3:**
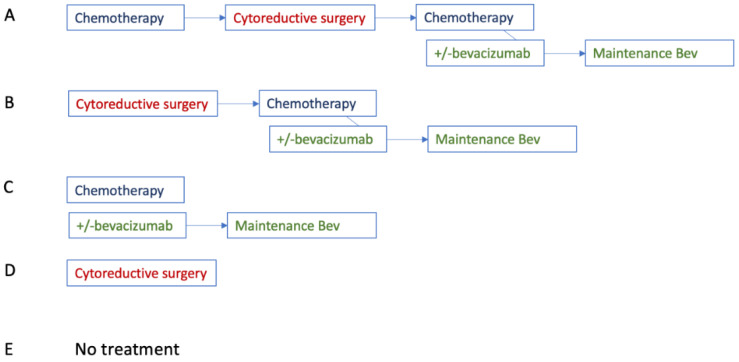
Treatment cohorts A–E.

Patients were sorted into the following 5 pre-defined treatment cohorts: (A) neoadjuvant chemotherapy (NACT) followed by interval cytoreductive surgery; (B) primary cytoreductive surgery (PCS) followed by adjuvant therapy; (C) systemic therapy only. The remainder who did not receive systemic treatment were categorized as (D) surgery without chemotherapy; (E) neither surgery nor chemotherapy (Appendix E Figure A3).

**Figure A4:**
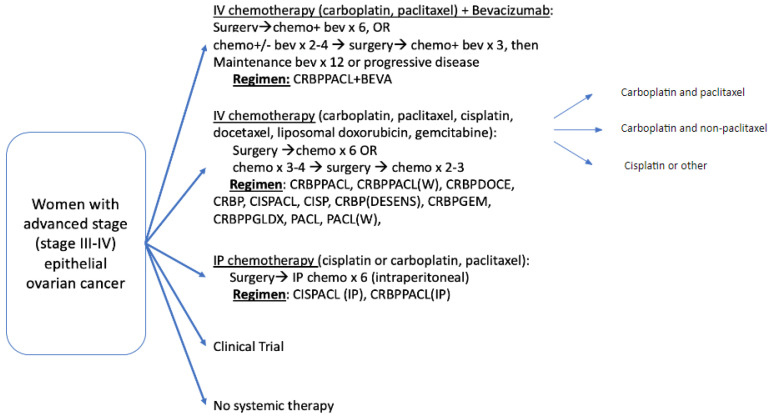
Identification of first-line regimen using ALR and NDFP.

Chemotherapy was identified from the ALR and NDFP databases. Regimen was assigned based on first cycle of chemotherapy (Appendix E Figure A4). Regimen protocol that contained bevacizumab in the first-line setting, whether it was used in the first cycle or not, was included. The regimen list was individually reviewed to exclude supportive care regimens and regimens for other malignancies. Analyses on patients receiving bevacizumab were restricted to the years 2016 and beyond as bevacizumab became publicly funded in Ontario in April 2016.

**Figure A5:**
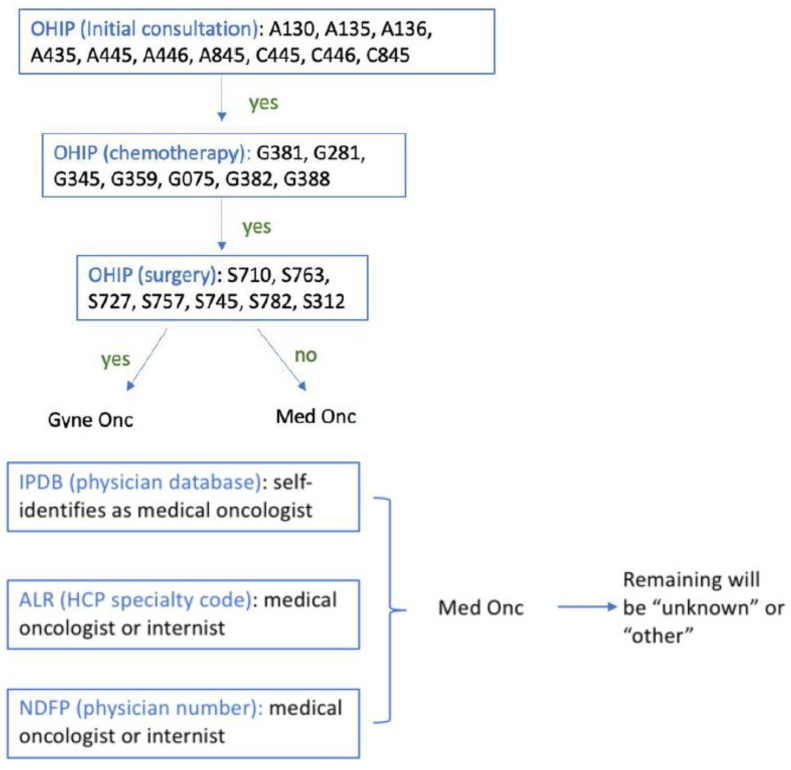
Definitions of providers of systemic therapy.

**Figure A6:**
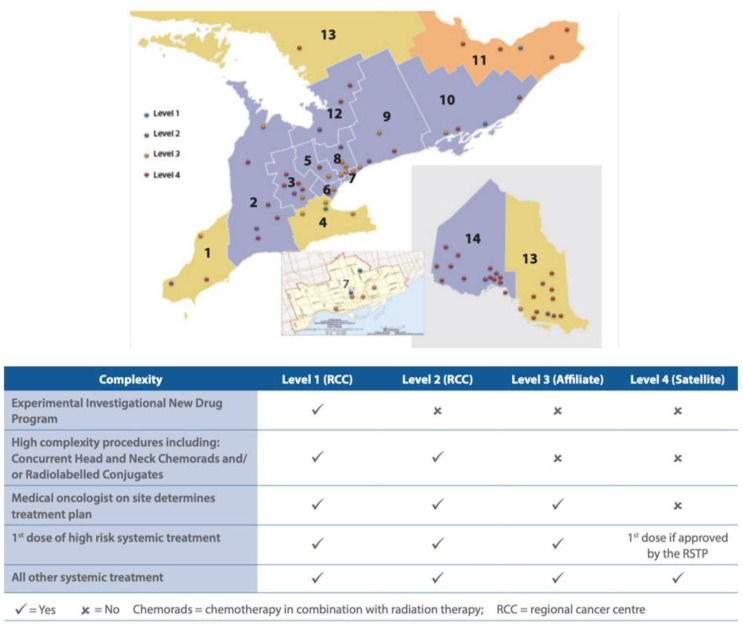
Definitions of facility levels for delivery of systemic therapy. ©Cancer Care Ontario Systemic Treatment Provincial Plan 2014–2019 [16] (permission obtained from corresponding author).

The providers of systemic therapy (gynecologic oncologist versus medical oncologist) were identified using OHIP physician billing codes (Appendix F Figure A5). The institution levels for the centre of first-line systemic therapy were assigned from ALR submitting hospital information, using the standardized designators as per Cancer Care Ontario (CCO) standard of level of facility for delivery of systemic therapy [16] (Appendix F Figure A6 and Table A5). In Ontario, there are 4 facility levels for the purpose of systemic therapy delivery in cancer care. Level 1 and 2 facilities are considered integrated cancer centres, with level 1 facilities capable of conducting clinical trials and academic teaching. In general, level 3 (affiliate) and 4 (satellite) facilities are considered community hospitals [17]. Gynecology oncology centres (GOC) where gynecologic oncologists practise and perform cytoreductive surgeries were also identified separately.

### 2.4. Explanatory Variables

Baseline demographic information included age at diagnosis, income quintile (obtained from Census), rurality score, and Charlson comorbidity score. For rurality score, a combination of rurality index for Ontario and a rural variable based on postal code were used to determine whether a patient lived in a rural residence [18]. Additional clinical variables included date of diagnosis (referred to as index date, which was obtained from OCR), date of surgery, date of death, type of surgeon, and surgical institution. 

### 2.5. Outcome Variables

**Figure A7:**
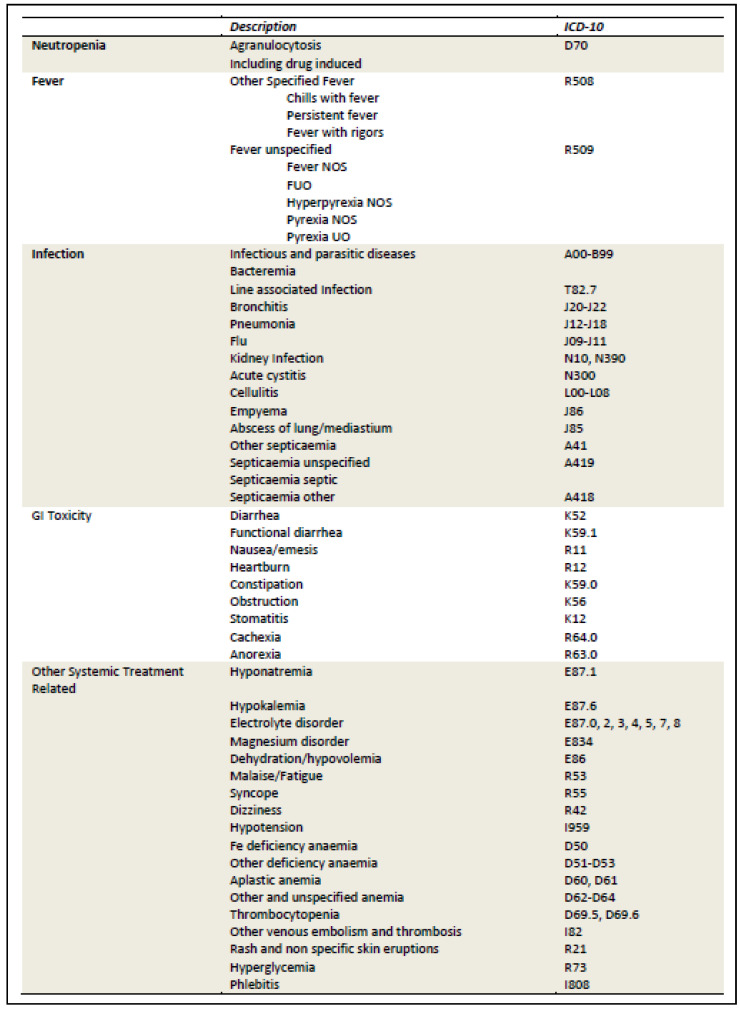
List of ICD-10 codes reflecting chemotherapy related toxicities. Adapted from Enright et al., *J. Oncol. Pract*. **2015**, *11*, 126–132 [19] (permission obtained from corresponding author).

Acute care utilization included emergency department (ED) visits and hospitalizations from the start of chemotherapy until the end of chemotherapy plus 30 days to account for the toxicity window following last treatment. ICD-10 codes were used for ED and hospital admission diagnoses using NACRS and CIHI-DAD respectively, limiting to main diagnosis when several were present. Reasons for ED visits and hospitalizations were categorized as potentially treatment-related if the associated primary diagnostic code was a common chemotherapy-related toxicity. A list of common chemotherapy-related toxicity was obtained based on previously developed and validated algorithms [19] (Appendix G Figure A7). The main diagnostic codes associated with ED and hospital admission in those who received bevacizumab were then individually reviewed and reclassified if it was deemed potentially related to the anti-angiogenic agent, based on a priori knowledge of bevacizumab associated toxicities. Finally, cancer-related diagnoses leading to ED visits or hospitalization were defined as all diagnostic codes related to ovarian cancer per OCR definition using ICD-10 codes (Appendix B Table A2).

### 2.6. Statistical Analyses

Descriptive statistics were used for the characterization of first-line treatment patterns, using Fisher’s exact test for comparisons when applicable with an alpha of <0.05 reflecting statistical significance. Multivariable logistic regression models using log-rank test was used to evaluate (1) bevacizumab use between gynecologic oncologist and medical oncologist and (2) bevacizumab use in tertiary academic (level 1) and non-tertiary centres (level 2, 3 and 4). Co-variates in the models were preselected to include age, Charlson score, rurality score. Stage (III vs IV) was not a co-variate as it cannot be differentiated using administrative data. Odds ratios and their 95% confidence interval were calculated. Date of death and follow-up were obtained to perform survival analyses for each treatment cohort. Due to lack of information on disease progression or recurrence using administrative databases, analyses on time to subsequent therapy and PFS could not be obtained. Survival analyses for OS were performed using Kaplan–Meier methods. All analyses were performed using SAS v9.4 (SAS Institute Inc., Cary, NC, USA).

## 3. Results

### 3.1. Baseline Characteristics

A total of 3726 patients met the inclusion criteria. Baseline demographics are shown in Table 1. The median age of the cohort was 67 (range 20–100). The majority had Charlson comorbidity scores of 0–2 (97.5%) and resided in urban areas (91%). Most histology codes reflected serous carcinoma (64%), although grading information was not available. 

### 3.2. Patterns of First-line Systemic Therapy

**Figure 1:**
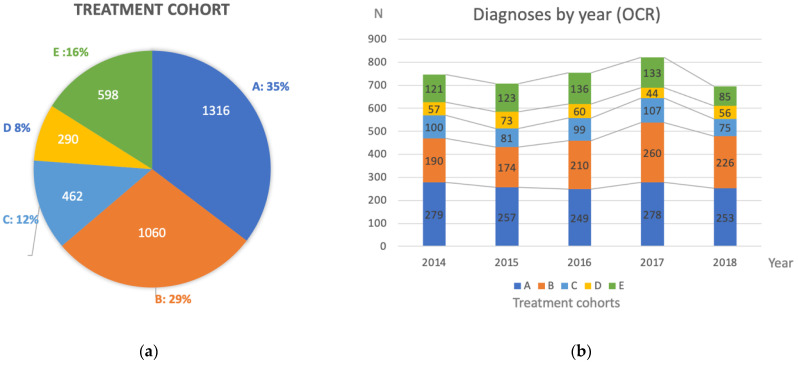
(**a**) Breakdown of predefined treatment cohorts; (**b**) diagnoses by year and treatment cohort. A: Neoadjuvant therapy followed by surgery; B: upfront surgery followed by chemotherapy; C: chemotherapy only; D: surgery only; D: no chemotherapy nor surgery.

A total of 2838 patients (76.2%) received chemotherapy in the first-line setting (Figure 1a). Of those, 1316 (46.3%) received NACT (Cohort A); 1060 (37.3%) underwent PCS (Cohort B); 462 patients (16.2%) received chemotherapy only (Cohort C). The remainder of 888 (23.8%) patients (Cohort D and E) did not receive systemic therapy (Appendix H Table A6). On average, 745 patients were diagnosed each year with advanced stage EOC in Ontario during the study period. (Figure 1b).

**Figure 2:**
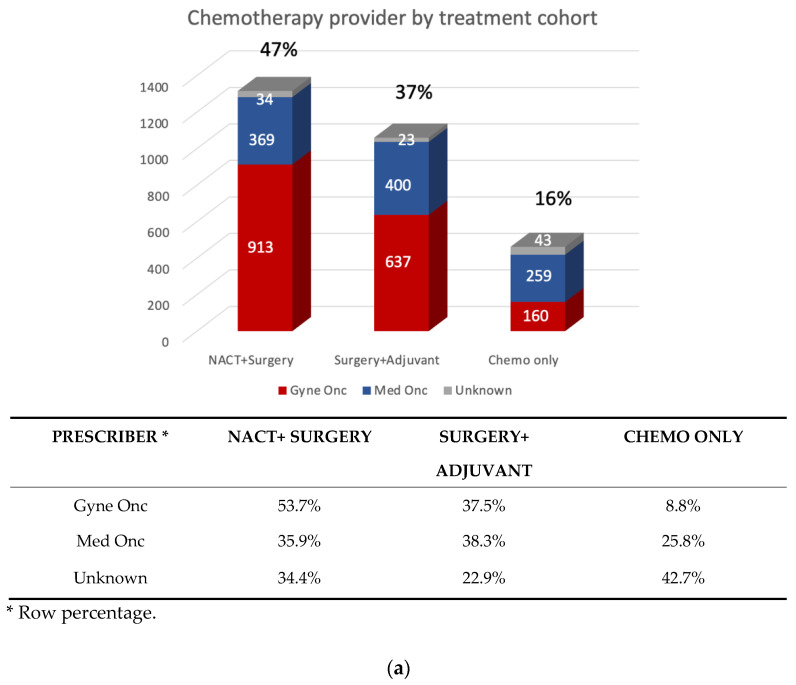

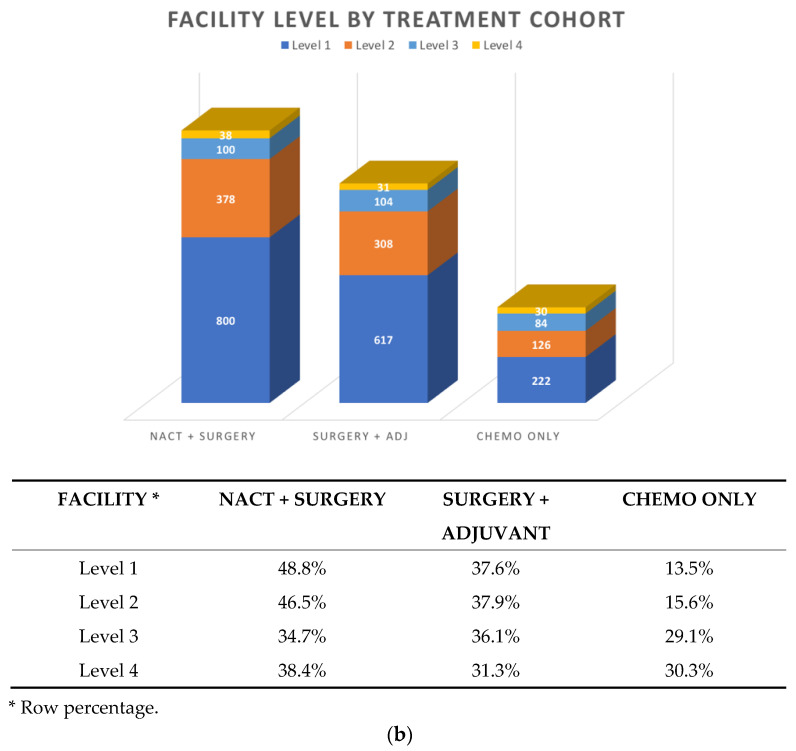
(**a**) Chemotherapy provider by treatment cohort.; (**b**) Facility level by treatment cohort.

Cytoreductive surgeries were performed by gynecologist oncologists in 2340 patients (71.7%). The first cycle of chemotherapy was most commonly prescribed by a gynecologic oncologist (1710, 60.2%) (Figure 2a). There was no significant difference in the prescription of upfront chemotherapy (cohort A and C combined) compared to adjuvant chemotherapy between gynecologic oncologists and medical oncologists (*p* = 0.677). Most cohort C patients received chemotherapy prescribed by a medical oncologist (256, 56.8%).

Most patients (1639; 58%) received chemotherapy in level 1 facilities, followed by level 2 (812, 29%), level 3 (288, 10%), and level 4 (99, 3%) (Figure 2b). Most level 1 facility chemotherapy was prescribed by gynecologic oncologists (1384, 84%), while most of the chemotherapy delivered in levels 2, 3, and 4 was prescribed by medical oncologists (Table 2). In comparison, GOCs were the surgical centres of 1348 (82.2%) patients treated with chemotherapy in level 1 facilities, 594 (73.1%) in level 2, 147 (51.0%) in level 3, and 60 (60.5%) in level 4. 

**Figure A8:**
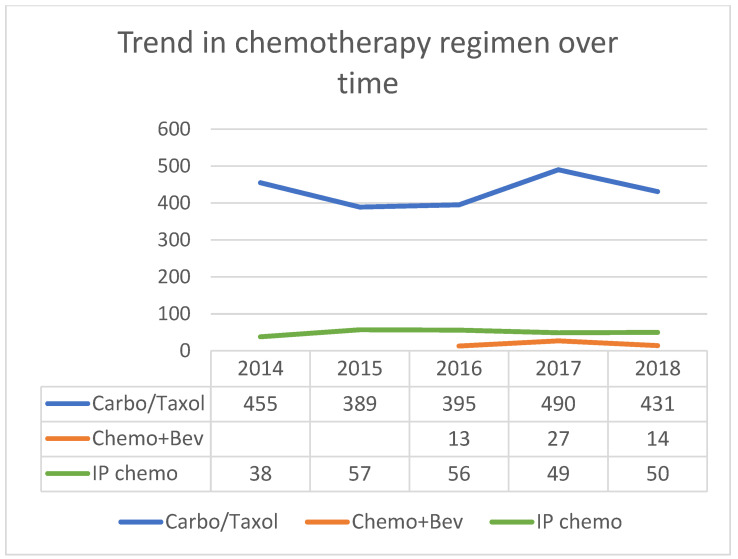
Chemotherapy regimen by year.

Most patients (2160; 76.1%) received intravenous carboplatin and paclitaxel chemotherapy. Restricting to 2016 and beyond, 54 patients (3.1%) received chemotherapy in combination with bevacizumab in the first-line setting as follows: 30 after NACT, 16 after PCS, and 8 without cytoreductive surgery. Bevacizumab was prescribed by medical oncologists in 37 (68.5%) patients and by gynecologic oncologists in 16 (29.7%) patients (*p* < 0.001). After adjusting for the cofounder facility and the predefined variables age, Charlson score, and rurality score, bevacizumab was four times more likely to be prescribed by a medical oncologist in the first line setting for advanced EOC compared to gynecologic oncologists (OR 3.95, 95% CI 2.11–7.14). The median duration of maintenance bevacizumab was seven months (ranging from six to eight). A total of 250 patients (8.8%) received IP chemotherapy. Of those, 226 (90%) were prescribed by gynecologic oncologists and 212 (85%) were delivered in level 1 facilities, higher than non-level 1 facilities (*p* < 0.001) (Appendix I Table A7 and Figure A8).

### 3.3. Acute Care Utilization during First-Line Treatment

During the predefined treatment period, there were 1561 patients (55%) with at least one emergency department (ED) visit and 1594 (56%) patients with at least one hospital admission. A total of 3338 ED visits occurred, 1302 (39%) of which were considered potentially treatment related. The most common main diagnoses of ED visits (after removing cancer diagnoses) were bowel obstruction (144, 4.3%) and fever (138, 4.1%). There were 1080 (32%) admissions from ED. A total of 2,378 hospitalizations occurred during the same timeframe, half of which (1184) were coded as related to cancer diagnosis and 23% (553) were considered potentially treatment related. The most common main diagnoses for hospital admissions (after excluding those due to cancer diagnoses) were neutropenia (134, 5.6%) and bowel obstruction (108, 4.5%).

ED visits occurred in 51% of cohort A (675), 57% of cohort B (602), and 61% of cohort C (284). Compared to cohort A, more ED visits occurred in cohorts B (*p* = 0.004) and C (*p* = 0.002). Two or more hospital admissions per patient (accounting for admissions related to cytoreductive surgery) occurred in 9.6% of cohort A (127), 26.8% of cohort B (285) and 14.5% (67) of cohort C (*p* < 0.001 A vs B). Hospitalizations in those treated with IP chemotherapy occurred in 61 patients (24.4%).

**Figure 3:**
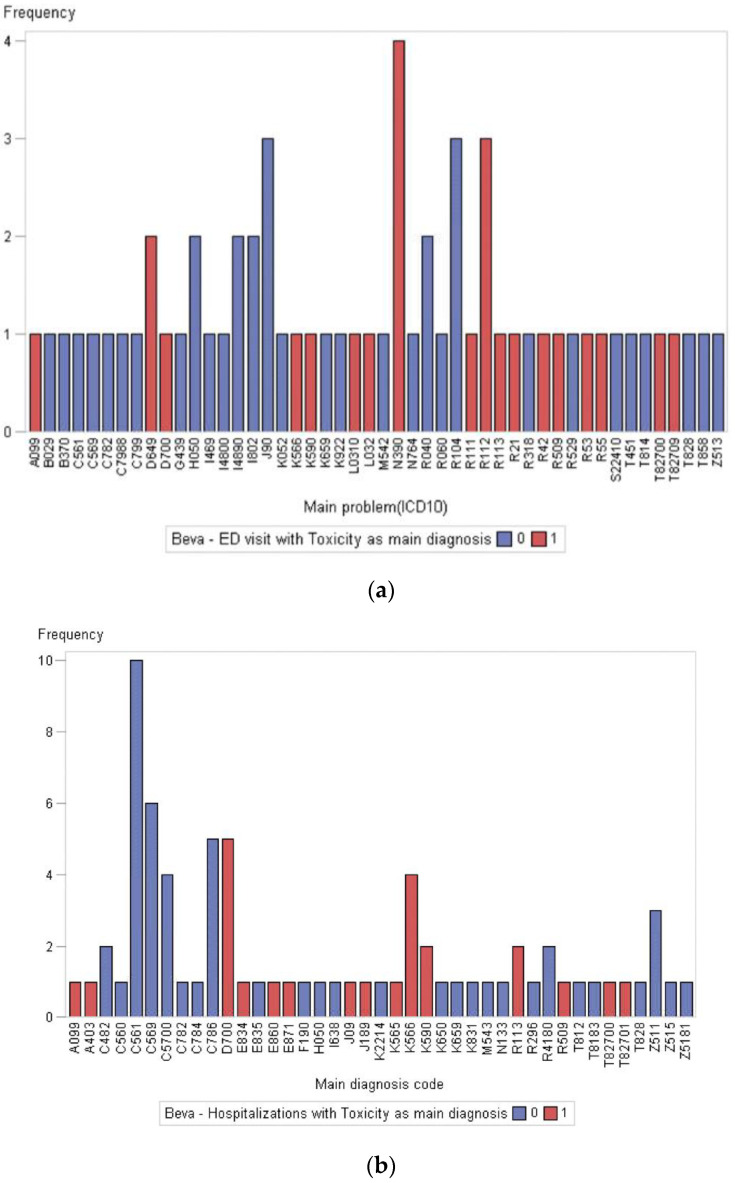
(**a**). ICD-10 codes of ED admission diagnoses during systemic treatment for patients receiving bevacizumab combination in first-line setting.; (**b**). ICD-10 codes for hospital admission diagnoses during systemic treatment for patients receiving bevacizumab combination in first-line setting. 0 = no; 1 = yes. For ICD-10 codes listed please refer to https://www.icd10data.com/ICD10CM/Codes (accessed on 11 February 2021)

Of 54 patients who received bevacizumab, 29 patients (53.7%) had at least one ED visit and 24 (44.4%) had at least one hospital admission. Among a total of 62 ED visits, 18 (29%) were considered treatment-related, and 14 (22%) were admitted to the hospital. The most common diagnoses for ED visits related to treatment toxicity were urinary tract infection and nausea and vomiting. A total of 13 (40%) hospitalizations occurred with cancer-related diagnoses and 7 (22%) hospitalizations were due to treatment-related diagnoses. There were no admissions for bowel perforation and fistulisation (Figure 3a,b).

### 3.4. Overall Survival

**Figure 4:**
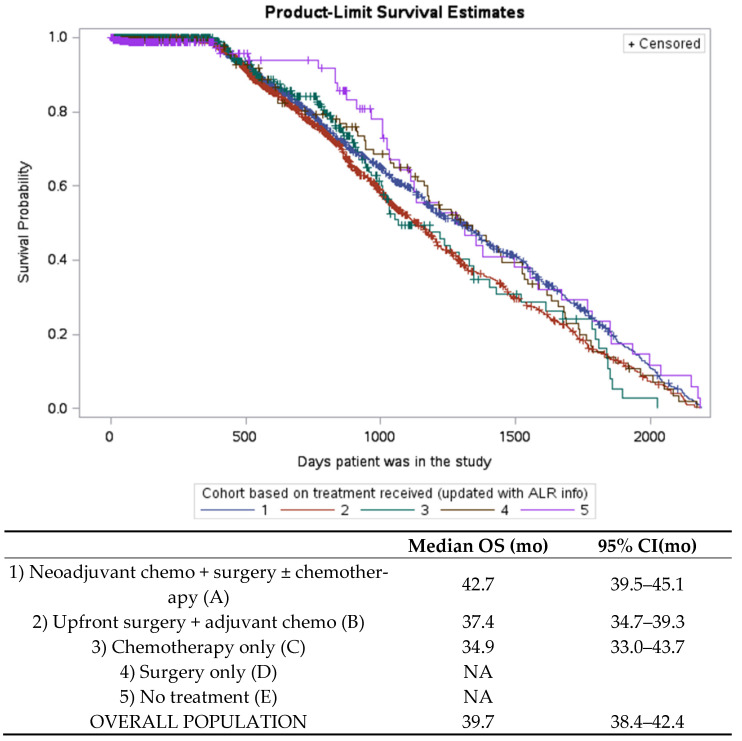
Kaplan–Meier survival analysis by treatment cohort. N/A-not assessed, given small numbers. OS-overall survival. 95% CI = 95% confidence interval. Mo-months.

The median OS in the entire cohort was 39.7 months (95% CI 38.4–41.4). The median OS in patients who underwent NACT was 42.7 months (95% CI 39.5–45.1) and the median OS in patients who underwent PCS followed by chemotherapy was 37.4 months (95% CI 34.7–39.3) (Figure 4).

## 4. Discussion

In this population-based cohort study evaluating real-world patterns of first-line systemic therapy including bevacizumab in advanced EOC in Ontario, we found that patterns of care were associated with physician specialty and that overall uptake of bevacizumab was low (3%). We saw a higher rate of bevacizumab use among medical oncologists, likely reflecting variations in referral patterns based on the stage and complexity of the patient. The majority of first-line chemotherapy was prescribed by gynecologic oncologists and in large academic tertiary cancer centres, in the context of the established Cancer Care Ontario organization for gynecology oncology services [20]. Acute care utilization during systemic therapy was high, with over half of patients having at least one ED visit and/or hospitalization during systemic therapy.

Several factors may explain the low adoption of bevacizumab in our cohort. First, implementation of a new policy in practice may take time after initial approval. Second, there may be hesitancy in prescribing bevacizumab in a population prone to bowel complications, especially among gynecologic oncologists who are also actively involved in the surgical aspect of patient care and are less likely to see patients with high-risk diseases (more likely to operate on stage III upfront). Third, prescribers may be concerned about the limited cost-effectiveness and the lack of a predictive biomarker to identify those patients most likely to benefit. Finally, as most Canadian jurisdictions only allow one line of therapy using bevacizumab, sequencing must be optimized, and oncologists may choose to use bevacizumab in the recurrent setting where the benefit has also been shown. A national survey of prescribers of systemic therapy for ovarian cancer would be helpful to better understand possible reasons and barriers to prescribing bevacizumab combination therapies in this setting.

Studies evaluating real-world use of NACT in EOC have shown substantial variation in utilization, ranging from 5% to 55% in high-volume hospitals in the United States in one recent study [21]. Nonetheless, data from patients treated in the United States prior to 2011 consistently show very low adoption of NACT, at less than 15% [22,23], with a trend toward an increase in the use of neoadjuvant treatment over time and improved survival outcomes for those treated with NACT compared to primary cytoreductive surgery [21]. In addition, there also seems to be an association between the use of NACT and patients with high-risk features, such as those with stage IV disease, older age, higher medical comorbidity, and poorer performance status [23], indicating a potential selection bias in all cohort studies comparing the use of NACT with PCS. A recent systematic review and meta-analysis of randomized trials, however, found no statistically significant difference in survival outcomes, including overall and progression-free survival, between patients treated with NACT and PCS [24]. More recently, another study using the National Cancer Database using linear modelling showed a larger decline in mortality in the liberal use of neoadjuvant chemotherapy compared to restrictive use [25]. Our results suggest a higher rate of NACT use, which is consistent with the current trend and practice, although any survival difference must be interpreted with caution due to inherent selection bias.

Studies evaluating differences in providers of chemotherapy for ovarian cancer have been scarce, as almost all the studies in the literature on physician specialty have focused on surgical specialization performing ovarian cancer surgeries [15,26,27]. One study using the SEER database of ovarian cancer patients treated two to three decades ago showed no difference in survival outcomes despite very different chemotherapy treatment styles [28]. It should be noted that systemic therapy options for ovarian cancer were quite limited at that time. Nonetheless, our results echo those findings and suggest ongoing variations in the choice of first-line systemic therapy regimen between medical and gynecologic oncologists. That being said, we agree with current practice guidelines recommending all advanced EOC patients be assessed by gynecologic oncologists prior to initiation of first-line treatment [3], as recent results using the ICES database have shown that this was associated with improvement in survival outcomes [29]. While most first-line chemotherapies were prescribed by gynecologic oncologists in the province of Ontario, many patients may subsequently be referred to medical oncologists in the recurrent setting as the number of lines of systemic therapy and options for clinical trials increase. With emerging new cancer therapies, including targeted therapies, and a rise in the complexity of cancer management, we believe the treatment of ovarian cancer should consistently take on a multidisciplinary approach to optimize patient care [30].

## 5. Limitations

There are several limitations to this study. Most importantly, without accurate staging information (stage III vs IV) and cytoreduction status, we cannot determine the appropriate denominator for the number of patients who would have been eligible for bevacizumab as this is only approved for those with high-risk disease (stage IV or suboptimal cytoreduction). Based on clinical experience treating ovarian cancer, we would expect a higher number of patients with high-risk disease and eligible for bevacizumab, including many patients in cohorts that did not receive surgery. In our study, only 54 patients, or 3%, received a bevacizumab-containing regimen in the first-line setting. As such, we believe the true adoption rate of bevacizumab remains low.

In addition, biases inherent in the retrospective nature of this study and large administrative data must be considered. As this is one of the first analyses of the ovarian cancer cohort at ICES, some of the variables have not been validated, while some of the databases may have missing data. Nonetheless, all these algorithms and protocols were developed and reviewed thoroughly by gynecologic oncologists and medical oncologists with expertise in ovarian cancer treatment. It would be prudent to undertake subsequent studies to specifically validate these algorithms.

Furthermore, despite high-quality data on ED visits and hospitalizations during treatment, we cannot accurately distinguish treatment-related toxicity from cancer-associated complications, especially for bowel-related complications. Moreover, while toxicity related to bevacizumab was relatively low, the small number of patients treated with bevacizumab and the short follow-up timeframe during treatment may not have captured all potential treatment-related toxicities. Overall, our data suggest there is a need to improve patient care in the ambulatory setting by using new tools and resources to reduce the acute care visits related to ovarian cancer. An ongoing intervention is the Multidisciplinary Ambulatory Management of Malignant Bowel Obstruction (MAMBO) program at the Princess Margaret Cancer Centre, which aims to utilize a multidisciplinary approach to manage malignant bowel obstruction for women with gynecologic malignancies (particularly ovarian cancer) in the ambulatory setting in order to reduce hospitalizations and improve patient outcomes [31].

Finally, we did not assess any PARP inhibitor-related data, an important aspect of targeted therapy in advanced EOC in the modern era, as oral PARP inhibitors were not publicly funded in the province during our study timeframe. In addition, the results of SOLO1 [7] were published in 2018, such that the timeframe of our study (2014–2018) did not contain PARP inhibitors in the first-line setting as a potential confounder. We also do not have biomarker and genetic information, including *BRCA* mutations, which can influence first-line treatment decisions for PARP inhibitors. More recently, with a clinical trial demonstrating the combination of PARP inhibitors and bevacizumab as a potential new first-line option for patients with a *BRCA* mutation [32], it would be interesting to see whether this will become another funded option in the first-line setting. Given the ever-changing landscape of ovarian cancer treatment, ongoing evaluation of patterns of care and associated real-world survival outcomes may be valuable.

## 6. Conclusions

In summary, patterns of care for first-line systemic therapy of advanced EOC in Ontario are heterogeneous amongst care providers and institutions. The overall adoption of bevacizumab for first-line treatment of advanced EOC has been low since its approval in Ontario. Physician and institutional factors leading to low uptake should be explored further. Ovarian cancer and cancer treatment-related acute care utilization is high and may benefit from further intervention. Given the complexity of patient care and the advances in systemic therapy, the management of ovarian cancer should continue to take a multidisciplinary team approach.

## Figures and Tables

**Table 1:** Baseline characteristics of patients with advanced EOC.

	N (%)
**Age**	Median: 67 Range: 20–100
**Charlson score** 0–2 ≥3	3634 (97.5%) 92 (2.5%)
**Rurality score** Urban Q1 * Q2 Q3 Q4 Q5 Rural	3396 (91.0%) 694 (20.4%) 708 (20.8%) 633 (18.6%) 641 (18.8%) 720 (21.2%) 330 (9.0%)
**Diagnosis year** 2014 2015 2016 2017 2018	747 (20%) 708 (19%) 754 (20%) 822 (22%) 695 (19%)
**Histology** Serous carcinoma Adenocarcinoma NOS Neoplasm NOS Carcinoma NOS Other	2395 (64.3%) 418 (11.2%) 314 (8.4%) 164 (4.4%) 435 (11.7%)
**Stage** Advanced (III and IV) Unknown	3586 (96.2%) 140 (3.8%)

* Q1–Q5: quintiles 1–5: The rurality income variable was calculated as such “to assess the relative impact of a rural primary residence location on outcomes, 20 without creating collinearity with the median income quintile, a hybrid variable incorporating both covariates were generated, termed “socioeconomic status (SES).” This is a six-level categorical variable, with all rural patients grouped into one category, and urban quintiles one to five representing increasing levels of median income. Using area-level data to impute individual SES has been described previously, and the resultant inferences appear valid” [18]. NOS = not otherwise specified.

**Table 2:** Chemotherapy prescriber by systemic therapy facility level.

Prescriber	Level 1	Level 2	Level 3	Level 4
Gyne Onc	1384 (84.4%)	285 (35%)	20 (7%)	21 (21%)
Med Onc	187 (11.4%)	512 (63%)	255 (88%)	74 (75%)
Unknown/Other	68 (4.2%)	15 (2%)	17 (level 3–4)	

Gyne Onc-gynecologic oncologist. Med Onc-medical oncologist.

## Data Availability

Restrictions apply to the availability of these data. Data was obtained from ICES and its linked datasets which are subject to ICES privacy and confidentiality agreements. Data can be requested from the corresponding author and obtained with the permission of ICES.

## Appendix A

**Table A1:** List of datasets accessed at ICES.

Dataset	Description	Use
Activity Level Reporting (ALR)	This is the main database on systemic therapy for cancer care in Ontario, which became robust as of 2014	Determine first-line regimen, hospital site, dates of treatment
Discharge Abstract Database (DAD) *	This captures demographic, clinical and administrative information on hospital admissions and discharges, including death.	Hospitalizations related to treatment toxicity during systemic therapy
ICES Physician Database (IPDB)	This contains information on physician specialty for those who provided this information	Determine physician type
National Ambulatory Care Reporting System (NACRS) *	This captures data for all hospital and community-based ambulatory care including emergency department visits and day surgery.	Determine ED visits and main diagnosis at ED related to toxicity during treatment
New Drug Funding Program (NDFP)	This contains all records of provincially funded drugs through Cancer Care Ontario	Determine receipt of bevacizumab
Ontario Cancer Registry (OCR)	This is the main registry for all cancer diagnoses in Ontario	Diagnosis and staging, histology and topography
Ontario Health Insurance Plan (OHIP)	This records all claims for physician reimbursement of inpatient and outpatient visits, consultations and procedures	Determine physician type, surgical type, referrals and consultations
Registered Persons Database (RPDB)	This data provides demographic information including health care card number, date of birth, sex and address	For baseline demographics including age and location
Same Day Surgery (SDS)	This records ambulatory visits for day surgeries	For ovarian cancer surgeries

* Using Canadian Institute of Health Information (CIHI).

## Appendix B

**Table A2:** ICD-10 codes included for ovarian cancer diagnoses.

	Description
C56 C560 C561 C569	Malignant neoplasm of the ovary Malignant neoplasm of the ovary, unilateral Malignant neoplasm of the ovary, bilateral Malignant neoplasm of the ovary, unspecified unilateral/bilateral
C570 C5700 C5701 C5709	Malignant neoplasm of fallopian tube Malignant neoplasm of fallopian tube, unilateral Malignant neoplasm of fallopian tube, bilateral Malignant neoplasm of fallopian tube, unspecified unilateral/bilateral
C48 C481 C482 C484	Malignant neoplasm of peritoneum Malignant neoplasm of specified part of peritoneum Malignant neoplasm of peritoneum, unspecified Neoplasm of uncertain or unknown behaviour of peritoneum

**Table A3:** ICD-O codes included for high grade histologies.

ICD-O Code	Description
80003 80013 80053 80103 80203 80503 81403 82553 82603 83103 83233 83403 83413 83423 83813 84403 84413 84503 84603 84613 89403 89503 89803 90143	neoplasm, malignant tumor cells, malignant malignant tumor carcinoma NOS carcinoma, undifferentiated NOS papillary carcinoma NOS adenocarcinoma NOS adenocarcinoma of mixed subtypes papillary adenocarcinoma NOS clear cell carcinoma NOS mixed cell adenocarcinoma papillary carcinoma, follicular variant papillary microcarcinoma papillary carcinoma, oxyphilic cell endometrioid adenofibroma, malignant cystadenocarcinoma NOS serous cystadenocarcinoma NOS papillary cystadenocarcinoma NOS papillary serous cystadenocarcinoma serous surface papillary carcinoma mixed tumor, malignant NOS Mullerian mixed tumor carcinosarcoma NOS serous adenoarcinofibroma

## Appendix C

A total of 2059 patients were initially identified as stage III and IV in OCR during the study timeframe. There were 1711 patients with an unknown stage. After applying the algorithm, 1527 patients were categorized as having an advanced stage, and 140 remained as unknown. Therefore, the final number of patients categorized as an advanced stage in the study was 3726.

## Appendix D

**Table A4:** List of ovarian cancer surgical codes in OHIP and CIHI-CCI.

CIHI-CCI	OHIP
**Partial excision of uterus and surrounding structures:** RM.87.DA-GX - Endoscopic (laparoscopic) approach. RM.87.CA-GX - Per orifice (transvaginal) approach. RM.87.LA-GX - Open approach **Total excision of uterus and surrounding structures** RM.89.AA - Using combined laparoscopic and vaginal approach RM.89.CA - Using vaginal approach. RM.89.DA - Using endoscopic (laparoscopic) approach. RM.89.LA - Using open approach. **Radical excision of uterus and surrounding structures** RM.91.AA - Using combined laparoscopic and vaginal approach. RM.91.CA - Using vaginal approach. RM.91.DA - Using endoscopic (laparoscopic) approach. RM.91.LA - Using abdominal approach (includes modified radical hysterectomy o 1NK87- excision partial, small intestine o 1NQ87- excision partial, rectum o 1NM87- excision partial, large intestine o 1NM89- excision total, large intestine o 1OB89- excision total, spleen	**Debulking surgery** o S710: Total Abdominal Hysterectomy (TAH) + Omentum +Bilateral Salpingo-oophorectomy (BSO) o S763: Radical Hysterectomy + BSO o S727: Debulking o S757: TAH +/-BSO o S745: USO or BSO (unilateral or bilateral salpingo-oophorectomy) o S782: USO or BSO + omenteum **Extensive surgery for stage IV** o S213: Low anterior resection o S312: Laparotomy o S149: Ileostomy o S157: Colostomy o S167: large intestine o S171: Left hemicolectomy o S166: Right hemicolectomy o R905: Splenectomy

## Appendix E

First-line chemotherapy timeframe was defined as: Initiation between 6 months before surgery and up to 9 months following surgery. Completion defined as the date after which no further chemotherapy was delivered for at least 60 days, when there was a change in regimen during this timeframe, or when the “intention of treatment” variable changed from adjuvant to palliative, whichever came first.

## Appendix F

**Table A5:** List of facilities included and assigned facility level.

Facility No.	Facility Legal Name	Facility Level
981	Chatham-Kent Health Alliance	4
933	Windsor Regional Hospital	2
966	Bluewater Health	3
793	St. Thomas Elgin General Hospital	4
813	Huron Perth Healthcare Alliance	4
889	Wingham & District Hospital	4
890	Woodstock General Hospital Trust	4
936	London Health Sciences Centre	1
955	Grey Bruce Health Services	3
661	Cambridge Memorial Hospital	3
665	Guelph General Hospital	4
930	Grand River Hospital	2
963	North Wellington Health Care Corporation	4
718	Joseph Brant Hospital	3
942	Hamilton Health Sciences Corporation	1
962	Niagara Health System	2
970	Brant Community Healthcare System	3
916	Headwaters Health Care Centre	4
951	William Osler Health System	3
950	Halton Healthcare Services Corporation	3
975	Trillium Health Partners	2
976	Sinai Health System	3
980	Unity Health Toronto	3
947	University Health Network	1
858	Toronto East Health Network	3
953	Sunnybrook Health Sciences Centre	1
632	North York General Hospital	3
701	Mackenzie Health	3
736	Southlake Regional Health Centre	2
905	Markham Stouffville Hospital Corporation	3
941	Humber River Hospital	3
771	Peterborough Regional Health Centre	3
940	Northumberland Hills Hospital	3
952	Lakeridge Health	2
979	Scarborough Health Network	3
619	Brockville General Hospital	4
693	Kingston General Hospital	1
592	Lennox and Addington County General Hospital	4
928	Perth and Smiths Falls District Hospital	4
957	Quinte Health Care	3
763	Pembroke Regional Hospital Inc.	4
788	Renfrew Victoria Hospital	4
800	Hopital General de Hawkesbury & District General Hospital Inc.	4
882	Winchester District Memorial Hospital	4
967	Cornwall Community Hospital	4
958	The Ottawa Hospital	1
606	Royal Victoria Regional Health Centre	2
968	Muskoka Algonquin Healthcare	4
745	Orillia Soldiers’ Memorial Hospital	4
638	The Lady Minto Hospital	4
650	St. Joseph’s General Hospital Elliot Lake	4
687	Sensenbrenner Hospital	4
784	Manitoulin Health Centre	4
881	Hopital General de Nipissing Ouest/The West Nipissing General Hospital	4
888	Temiskaming Hospital	4
974	North Bay Regional Health Centre	4
681	Hôpital Notre-Dame Hospital (Hearst)	4
907	Timmins and District Hospital	4
696	Kirkland and District Hospital	4
931	West Parry Sound Health Centre	4
959	Health Sciences North / Horizon Santé Nord	2
965	Sault Area Hospital	3
600	Atikokan General Hospital	4
647	Dryden Regional Health Centre	4
662	Geraldton District Hospital	4
719	Manitouwadge General Hospital	4
977	North of Superior Healthcare Group	4
826	Lake of the Woods District Hospital	4
896	The Red Lake Margaret Cochenour Memorial Hospital Corporation	4
900	Riverside Health Care Facilities Inc.	4
935	Thunder Bay Regional Health Sciences Centre	2
964	Sioux Lookout Meno-Ya-Win Health Centre	4

## Appendix H

**Table A6:** Cohorts who did not receive systemic therapy.

	Cohort D (Surgery Only) *N* = 290	Cohort E (No Treatment) *N* = 598
Stage III or IV Missing	266 (92%) 24 (9%)	539 (90%) 59 (10%)
Age group Less than 70 70–79 80+	133 (45%) 90 (31%) 67 (23%)	140 (23%) 116 (19%) 342 (57%)
Histology Serous carcinoma Neoplasm, carcinoma or adenocarcinoma	138 (47%) 55 (19%)	88 (15%) 461 (77%)
Death during follow-up	212 (73%)	556 (93%)

## Appendix I

**Table A7:** Chemotherapy regimen breakdown.

Chemotherapy Regimen	Total N (%)	Providers		Facility Levels	
		Gyne Onc	Med Onc	Level 1	Level 2–4
**IV carboplatin + Taxol**	2160 (76.1%)	1286	808	1189	971
**IV carboplatin + non-Taxol**	341 (12%)	178	133	204	137
**IP chemotherapy**	250 (8.8%)	266	24	212	38
**IV chemotherapy + bev**	54 (1.9% *)	17	37	16	38
**Other (including cisplatin and trial)**	33 (1.2%)				

* after 2016.
